# Early pregnancy metabolic syndrome and risk for adverse pregnancy outcomes: findings from Rajarata Pregnancy Cohort (RaPCo) in Sri Lanka

**DOI:** 10.1186/s12884-023-05548-y

**Published:** 2023-04-05

**Authors:** Imasha Upulini Jayasinghe, Thilini Chanchala Agampodi, Ajith Kumara Dissanayake, Suneth Buddhika Agampodi

**Affiliations:** 1 Department of Community Medicine, Faculty of Medicine and Allied Sciences, Rajarata University of Sri Lanka, Anuradhapura, Sri Lanka; 2 Department of Gynaecology and Obstetrics, Faculty of Medicine and Allied Sciences, Rajarata University of Sri Lanka, Anuradhapura, Sri Lanka

**Keywords:** Metabolic syndrome, Pregnancy outcomes, Large for gestational age, Small for gestational age, Preterm birth

## Abstract

**Background:**

Despite the intergenerational effects of metabolic disorders, evidence is greatly lacking on early pregnancy metabolic syndrome (MetS) and its effects on pregnancy outcomes from low- and middle-income countries. Thus, this prospective cohort of South Asian pregnant women aimed to evaluate how early pregnancy MetS would affect pregnancy outcomes.

**Methods:**

A prospective cohort study was conducted among first-trimester (T1) pregnant women of Anuradhapura district, Sri Lanka recruited to the Rajarata Pregnancy Cohort in 2019. MetS was diagnosed by the Joint Interim Statement criteria before 13 weeks of gestational age (GA). Participants were followed up until their delivery, and the major outcomes measured were large for gestational age (LGA), small for gestational age (SGA), preterm birth (PTB) and miscarriage (MC). Gestational weight gain, gestational age at delivery and neonatal birth weight were used as measurements to define the outcomes. Additionally, outcome measures were re-assessed with adjusting fasting plasma glucose (FPG) thresholds of MetS to be compatible with hyperglycemia in pregnancy (Revised MetS).

**Results:**

2326 T1 pregnant women with a mean age of 28.1 years (SD-5.4), and a median GA of 8.0 weeks (IQR-2) were included. Baseline MetS prevalence was 5.9% (n = 137, 95%CI-5.0–6.9). Only 2027 (87.1%) women from baseline, had a live singleton birth, while 221(9.5%) had MC and 14(0.6%) had other pregnancy losses. Additionally, 64(2.8%) were lost to follow-up. A higher cumulative incidence of LGA, PTB, and MC was noted among the T1-MetS women. T1-MetS carried significant risk (RR-2.59, 95%CI-1.65–3.93) for LGA, but reduced the risk for SGA (RR-0.41, 95%CI-0.29–0.78). Revised MetS moderately increased the risk for PTB (RR-1.54, 95%CI-1.04–2.21). T1-MetS was not associated (p = 0.48) with MC. Lowered FPG thresholds were significantly associated with risk for all major pregnancy outcomes. After adjusting for sociodemographic and anthropometric confounders, revised MetS remained the only significant risk predictor for LGA.

**Conclusion:**

Pregnant women with T1 MetS in this population are at an increased risk for LGA and PTB and a reduced risk for SGA. We observed that a revised MetS definition with lower threshold for FPG compatible with GDM would provide a better estimation of MetS in pregnancy in relation to predicting LGA.

**Supplementary Information:**

The online version contains supplementary material available at 10.1186/s12884-023-05548-y.

## Background

Metabolic syndrome (MetS), a cluster of metabolic derangements, affects one-quarter of the global population [1, 2] and continues to rise with the global epidemic of obesity [3]. Dysglycemia, hypertension and atherogenic dyslipidemia which are pathogenically linked by central obesity, are the primary metabolic disturbances of concern in MetS [2]. The prevalence is reported to be higher among females [1, 2]. With the current trends in obesity, it is quite likely for a woman to have MetS, either diagnosed or undiagnosed, when she becomes pregnant. However, a consensus on a diagnostic definition for MetS in early pregnancy is not available, limiting the availability of proper estimates of its prevalence [4, 5]. Nevertheless, MetS holds a significant risk for metabolic diseases, including future type 2 diabetes mellitus and cardiovascular diseases [2]. The effects of MetS during pregnancy and on pregnancy outcomes are yet to be explored in Asian populations.

With compelling scientific evidence from David Barker on adult-onset metabolic diseases as a consequence of intrauterine and early-life exposure to stressful adverse environments [6], the Developmental Origins of Health and Disease (DOHaD) hypothesis is supported without contention. This concept is further implicated in numerous emerging scientific evidence on foetal programming, developmental plasticity, and epigenetic mechanisms where the developing foetus adapts itself to the future metabolic response by reprogramming its epigenome [7–10]. Furthermore, on the background of epigenetics, evidence shows that a metabolically disturbed maternal intrauterine environment directly affects the developing foetus and results in lifelong consequences related to metabolic diseases in the offspring [11–16]. Further, evidence suggests that these intrauterine exposures not only affect the immediate offspring but also have transgenerational epigenetic inheritance [17, 18].

Evidence from large scale longitudinal studies indicate that maternal obesity and metabolic status have direct effects on the offspring and its later life [19, 20]. Recent scientific evidence suggests that maternal MetS has effects on the telomere length of the neonates, predisposing the progeny to accelerated aging [21]. Also, studies among white, Caucasian pregnant women have provided evidence that MetS in pregnancy is associated with several adverse pregnancy outcomes [22–26] and that pathophysiologically deranged adipokine levels in MetS of these white women are predictors of adverse pregnancy outcomes [23, 27]. However, there is a paucity of evidence on how very early pregnancy metabolic derangements as well as composite MetS affect the course of pregnancy and its outcomes in other ethnic populations. This is important as different ethnic groups are at different levels of risk for cardio-metabolic diseases and observations from one ethnic population might not be applicable to another [28, 29]. Also, there is a scarcity of evidence on MetS in pregnancy from low- and middle-income countries.

On this context, the primary objective of our study was to evaluate how early pregnancy MetS would affect pregnancy outcomes in a prospective cohort of pregnant women from Sri Lanka. We also aimed to evaluate the association of individual metabolic parameters with the selected pregnancy outcomes of this population.

## Methods

### Study design and population

A community-based prospective cohort study, the Rajarata Pregnancy Cohort (RaPCo) [30], was conducted in Anuradhapura, the geographically largest district in Sri Lanka, commencing participant recruitment in July 2019. Study participants were newly registering pregnant women in all 22 Medical Officer of Health (MOH) areas of the Anuradhapura district. Baseline recruitment of the participants took place over three months via 226 RaPCo clinics in field settings. The RaPCo study enrolled over 90% of eligible newly registered pregnant women in Anuradhapura district, Sri Lanka, during the third quarter of 2019. The current study is a cohort analysis of the pregnant women from this large population-based RaPCo study.

The inclusion criteria adopted were pregnant women older than 18 years of their age and with singleton pregnancies in their first trimester (T1) (less than 13 gestational weeks). First trimester gestational age (GA) was determined using ultrasound scan (USS) data and for those without USS data, the last menstrual period (LMP) was used. Pregnant women being treated for asthma, hypothyroidism, autoimmune diseases, cardiovascular events (myocardial infarction and stroke), and those on steroids and hormonal therapies (as self-reported) were excluded from the study. A complete baseline assessment was performed for women recruited to identify those with and without ‘exposure’ (defined below). Participants were followed up until their delivery for pregnancy outcome assessment.

Since the study was based on the entire cohort data, sample size calculation was not done, but the power of the study was calculated retrospectively. Based on the numbers in exposed (137), and unexposed groups (2189), the power of the study to detect the association between MetS and large for gestational age (LGA) neonates, small for gestational age (SGA) neonates, and preterm birth (PTB) was 98.7%, 81.5% and 47.7% respectively.

### Baseline data collection

Baseline assessment included an interviewer-based, detailed clinical interview, anthropometric measures, and venepuncture for biochemical assays. The baseline sociodemographic and obstetric information included details on ethnicity, age at conception, education level, parity, and gravidity.

Anthropometric measurements included weight, height, waist circumference (WC), and body mass index (BMI) assessments performed according to the standard protocols. Blood pressure (BP) was measured in all participants using a digital blood pressure meter (OMRON HEM-7320). The biochemical evaluation included serum analysis for fasting plasma glucose (FPG), serum triglyceride (TG), total cholesterol (TC), low-density lipoprotein (LDL) and high-density lipoprotein (HDL) levels. A qualified nursing officer performed venipuncture under universal precautions. 2.5 mL of whole blood was collected into each Sodium fluoride/Potassium oxalate (NaF/K2C2O4) tube for FPG and plain tube for serum analysis for lipid profile. Following fasting blood collection, every mother except those with pre diagnosed diabetes mellitus (DM), were given 75 g of glucose dissolved in 300 ml of water and second venipuncture was done after 2 h to collect 2.5 mL of whole blood to NaF/K2C2O4 tube for 2 h oral glucose tolerance test (2 h OGTT). All the samples were labelled with serial code number and transported in a cool box within 4 h of collection to Public Health Research Laboratory of Faculty of Medicine and Allied Sciences, Saliyapura. Biochemical samples were analyzed by an automated Mindray BS-240 clinical chemistry analyzer. The baseline biochemical assessment was conducted in all participants at T1. 2 h OGTT was repeated at second trimester (T2) between 24–28 gestational age for all participants except who were pre-conceptionally diagnosed as having DM and who were diagnosed as having hyperglycemia in pregnancy (HIP) at baseline biochemical assessment and only the FPG assessment carried out in them.

“Exposure” for this prospective cohort study was defined as pregnant women with MetS diagnosed by the Joint Interim Statement of the International Diabetes Federation (IDF) Task Force on Epidemiology and Prevention; National Heart, Lung, and Blood Institute; American Heart Association; World Heart Federation; International Atherosclerosis Society; and International Association for the Study of Obesity criteria [31]. MetS was diagnosed if a pregnant woman had any three of the followings: central obesity (defined as WC ≥ 80 cm), raised TG ≥ 150 mg/dL (1.7 mmol/L) or specific treatment for this lipid abnormality, reduced HDL cholesterol < 50 mg/dL (1.3 mmol/L) or specific treatment for this lipid abnormality, raised blood pressure ≥ 130/85 mm Hg or the treatment of chronic hypertension and raised FPG ≥ 100 mg/dL (5.6 mmol/L), or previously diagnosed type 2 diabetes mellitus. Other than the metabolic derangements mentioned under the definition of MetS, TC value of ≥ 200 mg/dl and LDL value of ≥ 100 mg/dl were considered as deranged lipid parameters for the study.

For this study, the T1 and T2 hyperglycemia in pregnancy was explained according to the World Health Organization (WHO) guidelines which uses the International Association of Diabetes in Pregnancy Study Group (IADPSG) thresholds [32]. Gestational diabetes mellitus (GDM) was diagnosed if one or more of the following criteria were met: FPG = 92–125 mg/ dl and 2-h PG = 153–199 mg/dl following a 75 g OGTT. DM in pregnancy was diagnosed if one or more of the following criteria were met: FPG ≥ 126 mg/dl and 2-h PG ≥ 200 mg/ dl following a 75 g OGTT. GDM and DM were collectively labelled as HIP. The detailed protocol on baseline evaluation and glycemic status evaluation of the cohort is published elsewhere [33]. BMI of the participants were categorized according to the Asia Pacific Guidelines for BMI classification [34].

### Outcome data collection

All pregnant women with and without MetS were followed up until their delivery. Pregnancy outcome data was collected from hospital delivery data registers and pregnant mothers’ registers in all public health midwife areas and through telephone interviews with the mothers. Several methods of outcome data collection with cross-checking were involved in maintaining the data accuracy and capturing those who had miscarriages and pregnant women who had left the area. These minimal interaction data collection methods were practiced, as physical data collection of pregnancy outcomes in hospital settings which were included in the original protocol, was hampered by the COVID-19 pandemic.

The main outcome variables studied included miscarriages (MC), preterm birth, large for gestational age neonates, small for gestational age neonates, gestational weight gain (GWG), neonatal birth weight (NBW), GA at delivery and maternal weight at delivery. Birth weight, sex, and gestational age at delivery were documented to calculate the sex-specific birth weight centile (BWC) in each neonate. INTERGROWTH 21^ST^ standards and tools [35] were used to calculate BWC, as this is the most recent tool developed and validated in eight different geographical areas in the world, including South Asia. Large for gestational age was defined as a birth weight ≥ 90th percentile for a given GA, and SGA was defined as a birth weight ≤ 10th percentile for a given GA. Preterm birth was defined as live births before 37 weeks of gestation. Live births after 37 weeks of gestation were defined as term births (TB). MC was defined as spontaneous loss of pregnancy before the fetus reaches its viability (until 24 weeks of gestation). Lost to follow-up in the outcome data was defined as those who have defaulted from the cohort and in whom none of the outcome data was reported.

### Data analysis

The baseline characteristics of the participants were described using descriptive statistics. Mean values with standard deviation (SD) and median value with interquartile range (IQR) information were given for normally distributed and non-normally distributed data, respectively. Independent sample t-test and Pearson’s correlation test were used to analyze the associations of metabolic parameters with each pregnancy outcome. The chi-squared test of association was used to analyze associations between categorical variables related to socio-demographics, metabolic parameters and pregnancy outcomes. MetS exposure status was reported as prevalence with percentage and 95% confidence interval (CI). In order to achieve the objectives of the cohort study, the risk was calculated to analyze the association between exposure and outcomes. Relative risk with 95% CI was calculated to individual determinants considered.

Logistic regression analysis was carried out to determine the adjusted risk estimates. For the missing outcome data, multiple imputation was used to improve the robustness of the model. We hypothesized that the association between MetS and the selected pregnancy outcomes studied could be influenced by age at conception, ethnicity, gravidity, education level, and BMI categories. In addition, gestational diabetes mellitus and diabetes mellitus in pregnancy (referred to as hyperglycemia in T1) was also included in the model as one of the most important determinants of selected pregnancy outcomes. Since hyperglycemia in T1 could be having an interaction effect with MetS, we looked into the main effect and interactions before deciding on the final model (Regression model A). The adjusted Odds Ratios (OR) were reported with 95% CI.

MetS diagnosis criteria are defined for the general adult population. With the changes in glucose metabolism early in pregnancy, normal plasma glucose levels differ from the non-pregnant population. This is the reason for having lowered thresholds for GDM. Similarly, the threshold for MetS diagnosis in pregnancy should also use lower thresholds. Based on this hypothesis, we tested a MetS definition with FPG 92 mg/dl for pregnancy for the regression model (Regression model B) adjusted for all above-mentioned covariates.

Since the original RaPCo showed a strong association between first trimester hyperglycemia with the risk of having large for gestational age (LGA) neonates [33], in this study we evaluated the association between MetS and pregnancy outcomes with having T1 hyperglycemia (GDM/DM) as a covariate. However, second trimester hyperglycemia generally considered a major risk factor for LGA and the effect or interaction of this factor with MetS requires cohort data with first and second trimester glucose assessment. COVID-19 pandemic prevented the complete data collection during the second trimester in this cohort. However, a subsample analysis was carried out among whom serum glucose values in both T1 and T2 was available to look at the unconfounded effect of MetS on LGA after adjustment for GDM.

Statistical Package for Social Sciences (SPSS) version 28 was used in data analysis. In all analytic results, statistical significance was considered as *p* < 0.05.

### Ethical considerations

Ethical clearance for the study was obtained from the Ethics Review Committee of the Faculty of Medicine and Allied Sciences, Rajarata University of Sri Lanka (ERC/2019/07). All the recruited pregnant women participated in the cohort study following the provision of informed written consent as well as data sharing, and publication consent.

This study is reported as per the Strengthening the Reporting of Observational Studies in Epidemiology (STROBE) guideline as shown in the additional file (see Additional file 1).

## Results

### Cohort characteristics

**Fig. 1:**
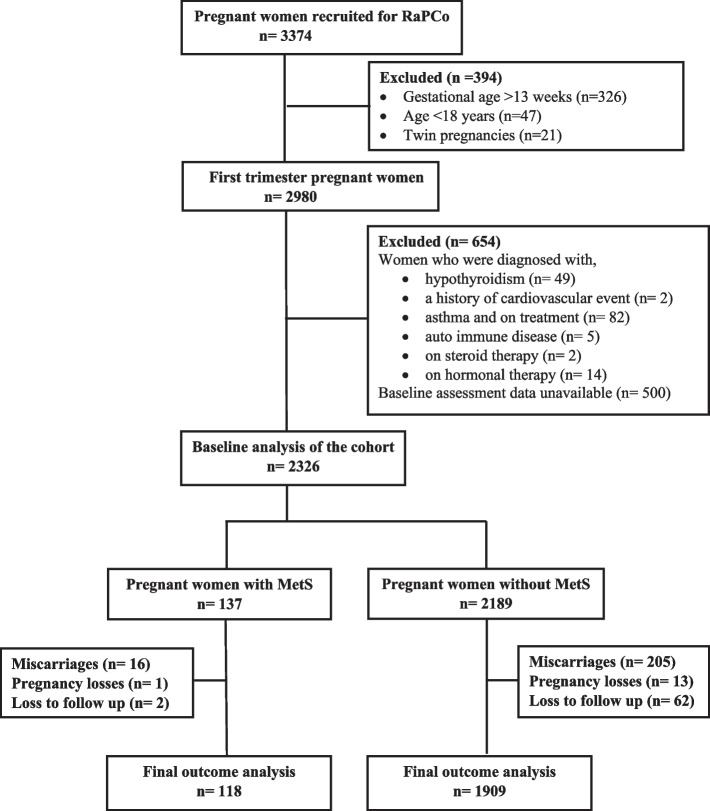
Participant flow in the cohort study

We included 2326 pregnant women from the original cohort using the eligibility criteria defined (Fig. 1). Their mean age was 28.1 years (SD 5.4), and the median and mean GA at recruitment was 8.0 weeks (IQR 2) and 8.2 weeks (SD 1.8), respectively. In the study sample, USS data for GA was available only in 40.5%, and in 50.5%, GA was calculated using the participants’ LMP. In both these groups, the median GA at recruitment was 8.0 weeks. In this study population, the prevalence of MetS at baseline was 5.9% (*n* = 137, 95%CI- 5.0–6.9). The revised MetS definition (FPG cutoff value lowered to diagnostic value of hyperglycemia in pregnancy) detected 180 (7.7%, 95% CI- 6.7- 8.9) pregnant women as having MetS.

Detailed baseline sociodemographic characteristics and metabolic parameters of the study participants are given in a previous cross-sectional study [5]. The sociodemographic characteristics and the metabolic parameters among the women with and without MetS in this study population are summarized in Table 1. In MetS and non-MetS groups, the number of women with preconceptionally diagnosed type 2 DM, hypertension and dyslipidemia 20 (14.6%) vs 15 (0.7%), 10 (7.3%) vs 54 (2.5%) and 04 (2.9%) vs 23 (1.1%) respectively. The differences observed in terms of sociodemographic, anthropometric, and metabolic parameters between the women with and without MetS were statistically significant (*p* < 0.05) except for GA at recruitment (*p* = 0.74), maternal education (*p* = 0.76), and height (*p* = 0.94). Table 2 summarizes the proportions of participants within each metabolic derangement defining the metabolic syndrome.

**Table 1:** Characteristics of the study cohort

	**Study population (*n* = 2326)**		**With MetS (*n* = 137)**		**Without MetS (*n* = 2189)**		***P* value**^**#**^
**Characteristic**	**n**	**%**	**n**	**%**	**n**	**%**	
*Ethnicity*							0.000
Sinhala	2048	88.1	99	72.3	1949	89.0	
Moor	251	10.8	33	24.1	218	10.0	
Tamil	22	0.9	5	3.6	17	0.8	
Other	5	0.2	0	0	5	0.2	
*Age at conception (years)*							0.000
< 20	131	5.6	3	2.2	128	5.8	
20–24	480	20.6	15	10.9	465	21.2	
25–29	839	36.1	35	25.5	804	36.7	
30–34	573	24.6	47	34.3	526	24	
35–39	259	11.1	31	22.6	228	10.4	
40–44	44	1.9	6	4.4	38	1.7	
*Gravidity*							0.004
1	703	30.2	27	19.7	676	30.9	
2	746	32.1	42	30.7	704	32.2	
3 or more	877	37.7	68	49.6	809	37.0	
*Parity*							0.035
00	786	33.8	32	23.4	754	34.4	
1	868	37.3	51	37.2	817	37.3	
2	568	24.4	43	31.4	525	24.0	
3	86	3.7	9	6.6	77	3.5	
4 or more	18	0.8	2	1.5	16	0.8	
*Gestational age at time of recruitment (weeks)*							0.742
4	35	1.5	3	2.2	32	1.5	
5	94	4.0	5	3.6	89	4.1	
6	278	12.0	13	9.5	265	12.1	
7	453	19.5	25	18.2	428	19.6	
8	503	21.6	27	19.7	476	21.7	
9	409	17.6	22	16.1	387	17.7	
10	261	11.2	20	14.6	241	11.0	
11	178	7.7	12	8.8	166	7.6	
12	115	4.9	10	7.3	105	4.8	
*Education level*							0.762
Up to GCE O/L	1372	59.0	81	60.9	1291	59.6	
Beyond GCE O/L	928	39.9	52	39.1	876	40.4	
*Metabolic parameters*							
Body mass index categories^*^							0.000
Underweight	383	16.5	0	0	383	17.5	
Normal	769	33.1	11	8.0	758	34.6	
Pre obese	364	15.6	15	10.9	349	15.9	
Obese class I	585	25.2	66	48.2	519	23.7	
Obese class II	225	9.7	45	32.8	180	8.2	
*Waist circumference*							0.000
≥ 80 cm	878	37.7	132	96.4	746	34.1	
< 80 cm	1448	62.3	5	3.6	1443	65.9	
	*Mean*	*SD*	*Mean*	*SD*	*Mean*	*SD*	
Weight (kg)	55.6	11.9	67.2	9.8	54.8	11.7	0.000
Height (cm)	154.1	5.8	154.1	6.4	154.1	5.7	0.942
BMI (kgm-2)	23.8	4.8	28.3	3.8	23.1	4.6	0.000
FPG (mg/dl)	82.3	12.8	99.7	27.3	81.2	10.4	0.000
TG (mg/dl)	87.1	38.3	143.8	50.1	83.6	34.4	0.000
HDL (mg/dl)	49.4	11.4	41.1	7.6	49.9	11.4	0.000
LDL (mg/dl)	122.5	32.1	141.4	33.6	121.3	31.6	0.000
TC (mg/dl)	171.9	34.4	182.4	35.3	171.3	34.3	0.000
SBP (mmHg)	102.8	11.5	111.0	12.8	102.2	11.3	0.000
DBP (mmHg)	65.4	8.2	71.2	9.9	65.1	8.0	0.000

*MetS* metabolic syndrome, *CI* confidence interval, *GCE O/L* general certificate of education ordinary level, *BMI* body mass index, *FPG* fasting plasma glucose, *TG* triglycerides, *TC* total cholesterol, *HDL* high density lipoproteins, *LDL* low density lipoproteins, *SBP* systolic blood pressure, *DBP* diastolic blood pressure, *WC* waist circumference, *SD* Standard deviation

^*^BMI categories are presented according to the Asia Pacific Guidelines for BMI classification

^#^Statistical significance was calculated to the *p* value of 0.05

**Table 2:** Study participants according to the metabolic derangements defined in metabolic syndrome

Metabolic derangement	With MetS (*n* = 137)		Without MetS (*n* = 2189)	
	N	%	N	%
FPG ≥ 100 mg/dL	61	44.5	37	1.7
HDL < 50 mg/dL	130	94.9	1137	51.9
TG ≥ 150 mg/dL	73	53.3	88	4.0
BP ≥ 130/85 mm Hg	25	18.2	33	1.5
WC ≥ 80 cm	132	96.4	746	34.1

*MetS* metabolic syndrome, *FPG* fasting plasma glucose, *TG* triglycerides, *HDL* high density lipoproteins, *BP* blood pressure, *WC* waist circumference

### Pregnancy outcomes

Out of 2326 women included in the baseline analysis, only 2027 (87.1%) women had a live singleton birth. MC was reported by 221 women (9.5%) and 14 (0.6%) women had other forms of pregnancy loss during the follow-up period. The percentage lost to follow up was 2.8% (*n* = 64). Among the deliveries, 99.9% were institutional deliveries and only one home delivery (0.1%) was reported. In terms of the mode of delivery 67.1% and 32.9% were vaginal and lower-segment caesarean sections, respectively. There were only 21 instrumental deliveries (forceps-05, vacuum-16). The median GA at delivery was 38.0 weeks (IQR 3). The average GWG of these women was 9.4 kg (SD 4.4). The mean NBW was 2938.9 g (SD 451.5). Moreover, 51.0% of neonates were males while 49.0% were females. Follow up indicated that 88.7% (n = 1795) of deliveries were term births while 11.3% (*n* = 229) were PTB. Among the women who had live births, BWC data was calculated only for 97.04%(1967) newborns, as there were 2.06% missing data for at least one of the three variables of NBW, sex, and GA at delivery. There were 143 (7.3%) LGA neonates and 351 (17.8%) SGA neonates. The mean BWC was 41.83 (SD 29.1). In this study population (*n* = 2326), the cumulative incidence of MC, LGA, SGA and PTB were 95.0, 61.5, 151, 98.5 per 1000 pregnant women, respectively.

### Metabolic parameters and pregnancy outcomes

In women who had a miscarriage (*n* = 221), the median GA at miscarriage was 11.0 weeks (IQR 2). Their mean BMI, WC, FPG, TG, HDL, SBP and DBP values at baseline were 23.9 Kgm^−2^ (SD 4.8), 78.3 cm (SD 12.1), 86.2 mg/dl (SD 18.9), 84.3 mg/dl (SD 39.8), 47.6 mg/dl (SD 11.5), 105.6 mmHg (SD 12.3) and 67.7 mmHg (SD 8.8) respectively. Only 16 (7.2%) of them had MetS in T1. Even though a higher incidence of miscarriage among those with MetS was observed (117 vs 94 per 1000 pregnancies), the presence of MetS in T1 was not significantly associated (*p* = 0.40) with having a miscarriage.

In the group with MetS (*n* = 137), only 118 (86.1%) had a live singleton birth while 16 women (11.7%) had MC, 01 (0.7%) woman had a pregnancy loss at GA 40 weeks, and 02 were (1.5%) lost to follow up. Among those without MetS, there were 1909 (87.2%), 205 (9.4%), 13 (0.6%) live births, miscarriages, and pregnancy losses, respectively, 62 (2.8%) were lost to follow-up. Among those with MetS, 67 (56.8%) and 51 (43.2%) had vaginal and cesarean deliveries respectively. In those without MetS, 1284 (67.8%) had vaginal deliveries and 610 (32.2%) had caesarean deliveries. Only a single (0.8%) vaginal instrumental delivery was reported among those with MetS while 20 (1.1%) instrumental deliveries were among non-MetS group. Comparison of measurements used to define the pregnancy and newborn outcome are summarized in Table 3.

**Table 3:** Comparison of measurements used to define the main pregnancy outcomes of the study (*n* = 2027)

Pregnancy outcome measure	With MetS (*n* = 118) Mean (SD)	Without Mets (*n* = 1909) Mean (SD)	Significance (Mean difference with 95% CI, *p* value)
Gestational weight gain (kg)	7.9 (3.8)	9.5 (4.4)	-1.6 [(-2.5) – (-0.8) < 0.001
GA at delivery (weeks)	37.8 (2.0)	38.3 (1.6)	-0.5 [(-0.8) – (-0.2)] 0.002
Birth weight (g)	3051.8 (533.9)	2931.8 (444.9)	120.4 (42.8–36.1) 0.005
Birth weight centile	53.4 (30.2)	41.1 (28.9)	12.3 (2.7 – 6.9) < 0.001

*GA* gestational age, *MetS* metabolic syndrome, *CI* confidence interval

NBW correlated with maternal WC (*r* = 0.16, *p* < 0.01), BMI (*r* = 0.14, *p* < 0.001), TG (*r* = 0.05, *p* = 0.03), and FPG (*r* = 0.09, *p* < 0.01) but not with the HDL, LDL, TC, and BP values of the mother in T1. In contrast, GWG was negatively correlated (*p* < 0.001) with maternal FPG (*r* = -0.08), TG (*r* = -0.12), TC (*r* = -0.1), LDL (*r* = -0.14), BMI (*r* = -0.23) and WC (*r* = -0.22) and positively correlated with HDL (*r* = 0.1, *p* < 0.001), but not correlated with maternal BP at T1. The mean GWG among pregnant women with deranged metabolic parameters of FPG, TG, HDL, LDL and WC were lower than the mean GWG of pregnant women with their normal metabolic parameters. But the mean GWG in women with deranged SBP and DBP were higher than that of pregnant women with normal BP parameters. Further analysis showed that these differences observed in the mean GWG were statistically significant (*p* < 0.001) in the TG, TC, HDL, LDL, FPG, and WC but not the BP measurements. Significant associations were observed for LGA with TC (χ^2^ = 6.6, *p* = 0.01), FPG (χ^2^ = 22.4, *p* < 0.001), WC (χ^2^ = 43.9, *p* < 0.001), BMI (χ^2^ = 50.8, *p* < 0.001), and TG (χ^2^ = 9.5, *p* = 0.002); for SGA with SBP (χ^2^ = 4.4, *p* = 0.04), FPG (χ^2^ = 10.0, *p* = 0.002), BMI (χ^2^ = 30.8, *p* < 0.001), and WC (χ^2^ = 36.3, *p* < 0.001); for MC with HDL (χ^2^ = 6.2, p = 0.012) and WC (χ^2^ = 8.1, *p* = 0.004) and for PTB with BMI (χ^2^ = 18.0, *p* = 0.001), and SBP (χ^2^ = 5.3, *p* = 0.021).

### MetS as a predictor of selected pregnancy outcomes

T1 MetS, irrespective of the definition, carried a significantly increased risk for LGA neonates (unadjusted RR 2.59 and 2.9 for MetS and revised MetS) but a reduced risk for SGA neonates (Table 4). Even though small, the risk for PTB was significant (RR = 1.52, 95% CI = 1.04–2.21) only with revised definition for MetS. It is noteworthy that the WC, as a single metabolic parameter was associated with all major pregnancy outcomes studied but PTB. Similarly, a lower FPG cutoff value was also associated significantly with all four major pregnancy outcomes. A higher cumulative incidence of LGA, PTB, and MC was noted among women with T1-MetS (Table 4).

**Table 4:** Incidence of selected pregnancy outcomes by first trimester MetS and individual metabolic derangements and their risk estimations

	**Pregnancy outcome**							
	**LGA**		**SGA**		**PTB**		**MC**	
	I (n)	RR(95%CI),*p* value	I (n)	RR (95%CI),*p* value	I (n)	RR (95%CI),*p* value	I (n)	RR (95%CI),*p* value
**MetS**								
Yes (*n* = 137)	145.9 (20)	**2.59** (1.65–3.93), ** < 0.001**	65.7 (09)	**0.41** (0.29–0.78), **0.006**	145.9 (20)	1.51 (0.98–2.3), 0.058	116.7 (16)	1.23 (0.76–1.98), 0.848
No (*n* = 2189)	56.6 (12)		156.2 (342)		95.5 (209)		93.7 (205)	
**Revised MetS**								
Yes (*n* = 180)	155.6 (28)	**2.9** (2.00- 4.27), < **0.001**	66.7 (12)	**0.4** (0.25- 0.74), **0.002**	144.4 (26)	**1.5** (1.04- 2.21), **0.031**	133.3 (24)	1.4 (0.97- 2.14), 0.068
No (*n* = 2146)	53.6 (115)		157.9 (339)		94.6 (203)		91.8 (197)	
**WC**								
≥ 80 cm (*n* = 878)	102.5(90)	**2.9** (2.1–4.0), ** < 0.001**	92.3 (81)	**0.5** (0.4–0.6), ** < 0.001**	113.9 (100)	1.3 (0.9–1.6), 0.05	117.3 (103)	**1.4** (1.1–1.8), **0.005**
< 80 cm (*n* = 1448)	36.6 (53)		186.5 (270)		89.1 (129)		81.5 (118)	
**BMI**								
≥ 23 kgm^−2^ (*n* = 1174)	87.7 (103)	**2.5** (1.8–3.6), < **0.001**	115.0 (135)	**0.6** (0.5–0.8), < **0.001**	110.7 (130)	**1.3** (1.0–1.6), **0.048**	103.1 (121)	1.2 (0.9–1.5), 0.12
< 23 kgm^−2^ (*n* = 1152)	34.7 (40)		187.5 (216)		85.9 (99)		86.8 (100)	
**FPG**								
≥ 100 mg/dl (*n* = 98)	173.5 (17)	**3.1** (1.9–4.8), ** < 0.001**	40.8 (04)	**0.3** (0.1–0.7), **0.006**	153.1 (15)	1.5 (0.9–2.5), 0.08	142.9 (14)	1.5 (0.9–2.5), 0.12
< 100 mg/dl (*n* = 2228)	56.6 (126)		155.7 (347)		96.1 (214)		92.9 (207)	
**FPG (GDM thresholds)**								
≥ 92 mg/dl (*n* = 248)	137.1 (34)	**2.7** (1.89- 3.85), < **0.001**	76.6 (19)	**0.5** (0.32–0.77), **0.002**	145.2 (36)	**1.6** (1.13- 2.18), **0.007**	129.0 (32)	**1.4** (1.01–2.02), **0.046**
< 92 mg/dl (*n* = 2078)	52.4 (109)		159.8 (332)		92.9 (193)		91.0 (189)	
**TG**								
≥ 150 mg/dl (*n* = 161)	118.0 (19)	**2.1** (1.3–3.2), **0.002**	111.8 (18)	0.7 (0.5–1.1), 0.12	130.4 (21)	1.4 (0.9–2.1), 0.14	93.2 (15)	0.9 (0.6–1.6), 0.97
< 150 mg/dl (*n* = 2165)	57.3 (124)		153.8 (333)		96.1 (208)		95.2 (206)	
**TC**								
≥ 200 mg/dl (*n* = 443)	85.8 (38)	**1.6** (1.1–2.3), **0.01**	128.7 (57)	0.9 (0.7–1.1), 0.23	112.9 (50)	1.2 (0.9–1.6), 0.22	103.8 (46)	1.4 (1.1–1.8), 0.01
< 200 mg/dl (*n* = 1883)	55.8 (105)		156.1 (294)		95.1 (179)		92.9 (175)	
**HDL**								
< 50 mg/dl (*n* = 1267)	68.7 (87)	1.3 (0.9–1.8), 0.09	142.1(180)	0.9 (0.7–1.1), 0.24	102.6 (130)	1.1 (0.8–1.4), 0.56	109.7 (139)	**1.4** (1.1–1.8), **0.01**
≥ 50 mg/dl (*n* = 1059)	52.9 (56)		161.5 (171)		93.5 (99)		77.4 (82)	
**LDL**								
≥ 100 mg/dl (*n* = 1692)	66.8 (113)	1.4 (0.9–2.1), 0.07	142.4 (241)	0.8 (0.7–1.0), 0.09	99.3 (168)	1.0 (0.8–1.4), 0.90	101.7 (172)	1.3 (0.9–1.8), 0.08
< 100 mg/dl (*n* = 634)	47.3 (30)		173.5 (110)		96.2 (61)		77.3 (49)	
**SBP**								
≥ 130 mmHg (*n* = 25)	80.0 (02)	1.4 (0.4–5.2), 0.63	00 (00)	-	240 (06)	**2.4** (1.2–4.9), **0.02**	200 (05)	2.1 (0.9–4.6), 0.07
< 130 mmHg (*n* = 2301)	61.3 (141)		152.5 (351)		96.9 (223)		93.9 (216)	
**DBP**								
≥ 85 mmHg (*n* = 44)	45.5 (02)	0.7 (0.2–2.8), 0.63	204.5 (09)	1.3 (0.7–2.4), 0.33	181.8 (08)	1.8 (0.9–3.5), 0.07	113.6 (05)	1.2 (0.5–2.7), 0.7
< 85 mmHg (*n* = 2282)	61.8 (141)		149.9 (342)		96.8 (221)		94.7 (216)	

*I* cumulative incidence per 1000 pregnant women, *n* total number of cases, *MetS* metabolic syndrome, *LGA* large for gestational age, *SGA* small for gestational age, *PTB* preterm birth, *MC* miscarriage, *FPG* fasting plasma glucose, *TC* total cholesterol, *TG* triglyceride, *HDL* high density lipoproteins, *LDL* low density lipoproteins, *SBP* systolic blood pressure, *DBP* diastolic blood pressure, *WC* waist circumference, *BMI* body mass index, *95%CI* 95% confidence interval

Regression analysis resulted in significant adjusted models for all outcomes with the exception of PTB (Table 4). After adjusting for possible confounders, T1 overweight/obesity and hyperglycemia remained as significant risk predictors for LGA neonates, but T1 MetS did not carry a significant risk. However, T1 MetS was a predictor of SGA neonates, but with a lowered risk (protective factor). Primi gravidity and T1 underweight were the significant risk predictors of SGA. After adjusting for confounders, T1 hyperglycemia remained a significant predictor only for LGA, however, there was no interaction effect of T1 hyperglycemia and MetS thus the interaction effect was removed from the model. Neither T1 MetS nor the T1 hyperglycemia were predictors of MC in this cohort. As the regression model A, regression model B also resulted in similar risk predictors for the pregnancy outcomes (Table 5).

**Table 5:** The adjusted effects of MetS on pregnancy outcomes

Outcome	Model	χ^2^(df), *p* value	PAC (%)	NR^2^	Significant predictor/s	OR	95% CI		*P* value
							Lower	Upper	
LGA	A	63.0 (9), < 0.001	93.7	0.071	Obesity^	2.29	1.47	3.55	< 0.001
					T1HG	2.06	1.40	3.03	< 0.001
					Age	1.05	1.01	1.09	0.016
	B^#^	64.3 (9), < 0.001	93.7	0.073	Obesity^^^	2.22	11.42	3.46	< 0.001
					T1HG	1.91	1.27	2.88	0.002
					Age	1.05	1.01	1.09	0.015
SGA	A	75.9 (9), < 0.001	83.4	0.054	MetS	0.48	0.24	0.99	0.046
					Primi gravida	1.80	1.29	2.52	0.001
					Underweight	1.56	1.16	2.10	0.003
					Obesity^^^	0.67	0.52	0.87	0.002
	B^#^	76.6 (9),,0.001	83.4	0.055	MetS	0.49	0.26	0.93	0.029
					Primi gravida	1.79	1.28	2.51	0.001
					Underweight	1.56	1.16	2.10	0.003
					Obesity^^^	0.68	0.52	0.88	0.004
MC	A	20.9 (9), 0.013	90.5	0.019	Age	1.05	1.01	1.08	0.005
	B^#^	22.2 (9), 0.008	90.5	0.020	Age	1.05	1.01	1.08	0.006
PTB^*^	A	13.5 (9), 0.140	90.1	0.012	None	-	-	-	-
	B^#^	13.4 (9), 0.146	90.1	0.012	None	-	-	-	-

*LGA* large for gestational age, *SGA* small for gestational age, *MC* miscarriage, *PAC* percentage accuracy in classification, *OR* odds ratio, *CI* confidence interval, *MetS* metabolic syndrome, *TG* triglyceride, *FPG* fasting plasma glucose, *WC* waist circumference, *DBP* diastolic blood pressure, *NR*^*2*^ Nagelkerke R^2^

A-Results of regression analysis with the model including MetS by joint interim statement, first-trimester hyperglycemia, BMI, and other sociodemographic factors (age, ethnicity, education, and gravidity)

B- Results of regression analysis with the model including MetS by revised definition, first-trimester hyperglycemia, BMI, and sociodemographic factors (age, ethnicity, education, and gravidity)

^*^All the models are insignificant for PTB

^#^Revised definition of MetS is the joint interim statement MetS definition with FPG cutoff value revised as ≥ 92 mg/dl

^^^The covariate “obesity”, includes pre-obese, obese class I and obese class II groups of BMI classification by Asia Pacific guidelines

### The completed cohort analysis with T2 GDM/DM

**Fig. 2:**
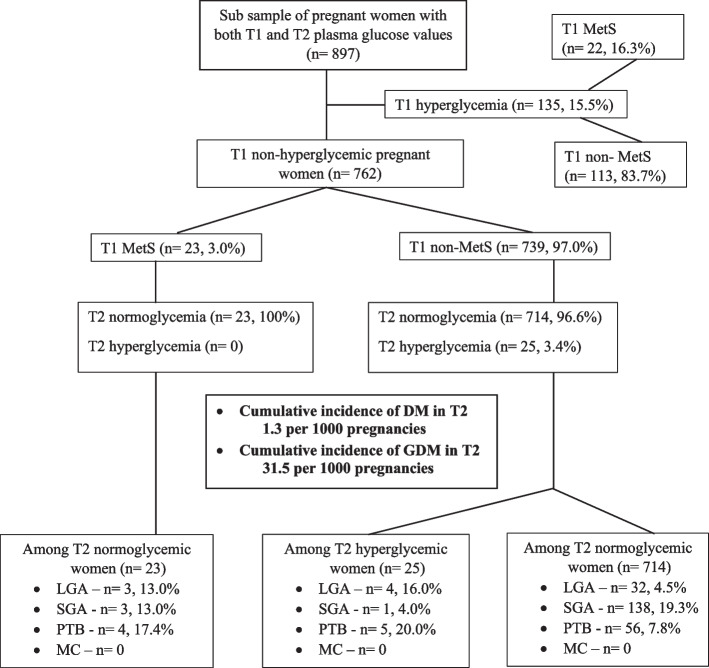
Sub sample analysis on incidence of hyperglycemia in second trimester. (T1- first trimester, T2- second trimester, MetS- metabolic syndrome, DM- diabetes mellitus, GDM- gestational diabetes mellitus, LGA- large for gestational age, SGA- small for gestational age, PTB- pre-term birth, MC- miscarriage. Hyperglycemia in pregnancy was defined according to the World Health organization criteria: GDM was diagnosed if one or more of the following criteria were met: Fasting Plasma Glucose (FPG) = 92–125 mg/dl and 2-h Plasma Glucose (2-h PG) = 153–199 mg/dl following a 75 g oral glucose tolerance test (OGTT). DM in pregnancy was diagnosed if one or more of the following criteria were met: FPG = 126 mg/dl and 2-h PG = 200 mg/ dl following a 75 g OGTT. GDM and DM were collectively labelled as hyperglycemia in this sample

A total of 897 pregnant women from the baseline cohort were participated in the second trimester data collection before the data collection was interrupted due to COVID-19 pandemic. For these pregnant women, both T1 and T2 GDM/DM assessment was available. Of them, 135 (15.05%) had either DM or GDM at T1 (Fig. 2). From the remaining 762 pregnant women, the incidence of GDM and DM at T2 were 31.5 and 1.3 per 1000 pregnancies, respectively. Among those who were normoglycemic at T1 but had MetS (n = 23), none of the pregnant women developed hyperglycemia at T2. Newly developed second-trimester GDM/DM was only apparent among T1 non-MetS pregnant women. Despite this observation, among women who had T1-MetS but without T1 or T2 hyperglycemia, the incidence of LGA (130 vs 45 per 1000 pregnant women) and PTB (174 vs 78 per 1000 pregnant women) were still high compared to women who did not have T1- MetS and hyperglycemia. Conditional logistic regression model including the same set of variables and T1/T2 hyperglycemia (DM/GDM) showed that only the model that used lowered FPG values for MetS diagnosis was significant. The adjusted MetS remained the only risk predictor (OR-2.53, 95% CI – 1.04- 6.16) for LGA while age, BMI, GDM/DM and other factors were not significant in this final model.

(Summary of the main results of the study cohort are provided in the Additional file 2).

## Discussion

In this study, we provide evidence on the associations between early pregnancy MetS and pregnancy outcomes in a rural South Asian population. Pregnant women with T1-MetS in this population carried a significant risk for having large babies, which will have an inter-generational effect on MetS associated health risks and events. It is also noteworthy that in this study, we tested a revised MetS criteria for diagnosing MetS in pregnancy based on the FPG thresholds for GDM, and this revised MetS seemed the best (and only) predictor of LGA. Other than having MetS as a composite factor, we also demonstrated how individual MetS parameters act as independent risk factors for selected pregnancy outcomes. We used a robust population based prospective cohort design with minimal selection bias, recruiting pregnant women very early in their pregnancy in comparison to previous studies published.

Unlike in the multicenter SCOPE study among white women, our study population of non-white origin had a higher relative risk for LGA, moderate risk for PTB, and a reduced risk for SGA. The SCOPE study finding that early pregnancy MetS was not associated with a risk of LGA, SGA and PTB in white women [22], was a similar finding to the first adjusted model of our study population. However, the subsample analysis with second trimester GDM data in our study shows that MetS is a significant predictor of LGA. In contrast to the relative risks for LGA in SCOPE study with raised glucose, we reported a significantly higher risk (RR 1.23 vs 3.1 and 2.7) with both cut-off values concerned for glucose levels. Furthermore, our subsample finding that none of the T1 MetS women developed T2 GDM/DM is contradictory to the findings from this white population who carried greatest risk with MetS for GDM incidence. This contradiction could also be due to the differences in sample sizes of the two populations. These observations may also be partly due to the ethnic predisposition in our study population, which was presumed to be at high risk for metabolic diseases [29]. On the other hand, the MetS assessment was done in the early second trimester in the SCOPE study(GA 15 ± 1 weeks), where the progressing gestation itself corresponded to changes in the metabolic profile starting at very early gestation [5], and the association may have been diluted.

Even though there were significant confounders, after adjustment for T2 GDM, T1 MetS had a higher risk for LGA. We believe that in this population, T1 MetS needs extensive and robust evaluation considering other important confounding factors for adverse pregnancy outcomes. Despite that, these finding are similar to the findings from one of the earliest studies evaluating T1-MetS and pregnancy outcomes [23]. This prospective study among white women with T1-MetS showed that they also had neonates with a significantly higher body weight [23]. This team of researchers also showed in their study group of Caucasian women with MetS, FPG and maternal BMI were significant risk factors for having LGA neonates [27]. Despite the difference in ethnic exposure, our study among South Asian pregnant women also showed that high levels of T1 FPG and high WC, and also the high BMI, carried nearly three times the risk for having LGA neonates. Unlike in our study, they also evaluated T1 serum adiponectin levels, which is an adipokine known to be reduced in the pathogenesis of MetS [36, 37], and found significantly decreased levels in both women with T1 MetS and women who had a large neonate [23, 27]. Also, two large scale prospective studies in Netherlands [38, 39] showed similar evidence as our finding in regard to a significant association of T1 TG levels with an increased risk for LGA (OR = 1.005 vs 1.11 and 1.48). In both these studies, they also showed that early trimester lipid levels were not significantly associated with SGA, which is an observation in our study cohort as well. Another recent Iranian study showed that early trimester TG levels significantly affect having a LGA neonate, even independently of GDM [40]. Together these support our finding of independently elevated risk for LGA in women with high T1 TG levels.

In the 2007 RHEA study in Greece [41], researchers showed that early pregnancy MetS carried nearly three times the risk for having a PTB, which was comparatively lower than the risk in our population (RR = 2.93 vs 1.40). However, in both these populations maternal blood pressure was a significant risk factor for PTB. Furthermore, a recent cohort study among Indian pregnant women that used ethnicity-specific BMI values showed that high BMI was significantly associated with an increased risk of both maternal and neonatal adverse outcomes, including MC, PTB and LGA [42]. Similarly, it was also evident in our study that overweight and obese women were at twice the risk of having a large baby if we are to consider T1 parameters. High BMI was also associated with increased risk for PTB. It is important to note that the elevated WC, which is a surrogate measure of central adiposity, significantly increased the risk for LGA, and MC of this population of pregnant women who were in their very early pregnancy, when it is unlikely to have their WC to be increased due to pregnancy. As in the Indian study which used ethnicity-specific Asian guidelines to stratify obesity, our cohort study also showed a significant association of ethnicity-specific WC with the adverse pregnancy outcomes. These similar findings from both the nations could be attributed to their common ethnic predisposition. Another Indian study [43] showed that early pregnancy hypertriglyceridemia in Indian women was significantly associated with PTB, but not the other lipid parameters. In contrast, our finding was that none of the lipid parameters were associated with the risk of PTB.

On a background of scarce evidence of a prospective association of very early pregnancy MetS with a number of pregnancy outcomes, a few studies on white pregnant women have shown that MetS, as a composite factor, increases the risk for GDM and hypertensive disorders in pregnancy [22–25, 44, 45]. However, such evidence is greatly lacking in other ethnic groups throughout the world. Several other studies that evaluated individual metabolic parameters in early pregnancy, with pregnancy outcomes showing that early pregnancy hypertriglyceridemia is significantly associated with GDM [24, 40, 43, 46, 47] as well as hypertensive disorders in pregnancy [43, 46, 48]. Another recent study in Brazil showed high TG, WC, FPG, and BP act as independent risk factors for hyperglycaemia in pregnancy [49]. In this particular population, T2 GDM/DM incidence was not evident among those who already had T1 MetS. The main reason for this observation was that we have evaluated the T1 glycemic status and classified T1 GDM using the WHO guidelines based on IADPSG criteria. It is evident that the majority of second trimester GDM could be diagnosed in T1 with IADPSG criteria. Among women who were having MetS (revised definition) and normoglycemic in T2 had 2.5 times the risk of delivering a LGA neonate showing that MetS has a significant independent effect on LGA. This observation also confirmed that the non-pregnant thresholds for MetS diagnosis may not be the best risk predictors in pregnancy. We have adjusted only the FPG threshold, but our previous work clearly shows gradual change of all metabolic parameters even within the first trimester and further studies on revising MetS thresholds to be used in pregnancy is recommended. Furthermore, consensus on proper diagnostic values is important to weigh the risk and benefits of pharmacological management of hyperglycemia in pregnancy for the offspring as well [50, 51].

Our outcome variables were also limited due to the effect of COVID-19 pandemic during the study period. The follow-up data collection was hampered by pandemic situation and quality data was lacking to comprehensively analyze the incidence and associations of hyperglycemia and hypertension in pregnancy with its outcomes in the whole cohort. The study also could not comprehensively analyze confounding effect by the treatments of hyperglycemia on pregnancy outcomes. This also resulted in lack of data to categorize PTB as either spontaneous or iatrogenic, hence, the study refers PTB to collectively the both. A comprehensive outcome profile assessment is required to understand the true effect of MetS in pregnancy together with other pregnancy related disorders. Also, the power calculation shows that the sample size may not be adequate for the evaluation of MetS on MC. Despite these limitations, this first population based large scale pregnant cohort in South Asia provides evidence which can be generalized to the populations in South Asia, who carries a distinct ethnic background with high risk for metabolic disease. Our findings might have limited applicability to white populations but would be useful in comparisons.

## Conclusions

In conclusion, we highlight that, South Asian pregnant women with early pregnancy MetS are at an increased risk for the adverse pregnancy outcomes of LGA neonates, and PTB as well as a reduced risk for SGA neonates. These findings are important for planning interventions, especially in LMIC populations, which are at critical stages of demographic, epidemiological, and nutrition transitions. We also showed that adjusting the FPG thresholds to define MetS to be compatible with GDM in pregnancy will provide a better estimation of MetS in pregnancy in relation to predicting LGA. Individual anthropometric and metabolic parameters also increased the risks for adverse pregnancy outcomes in this population. We further emphasize the necessity of robust, longitudinal research to evaluate the relationship between pregnancy MetS and its outcomes, particularly in diverse ethnic groups and populations, as well as the validity of MetS and its markers with confounders as predictors of adverse pregnancy outcomes.

## Supplementary Information

**Additional file 1.** 

**Additional file 2.**

## Data Availability

The data that support the findings of this study are available from the corresponding author upon reasonable request.

## Abbreviations

AHA/NHLBI: American Heart Association/National Heart, Lung and Blood Institute
BMI: Body Mass Index
CVD: Cardiovascular disease
DBP: Diastolic blood pressure
DM: Diabetes mellitus
FPG: Fasting plasma glucose
GDM: Gestational diabetes mellitus
HC: Hip circumference
HDL: High density lipoproteins
IDF: International Diabetes Federation
LDL: Low density lipoproteins
MetS: Metabolic syndrome
NCEP-ATP III: National Cholesterol Education Programme Adult Treatment Panel III
OGTT: Oral glucose tolerance test
POG: Period of gestation
SBP: Systolic blood pressure, SD, standard deviation
TC: Total cholesterol
TG: Triglycerides
T2DM: Type 2 diabetes mellitus
WC: Waist circumference
WHR: Waist hip ratio

## References

[1] Saklayen MG (2018). The Global Epidemic of the Metabolic Syndrome. Curr Hypertens Rep.

[2] International Diabetes Federation. The IDF consensus worldwide definition of the metabolic syndorme. International Diabetes Federation, 2006. 2006. p. 27.

[3] O’Neill S, O’Driscoll L (2015). Metabolic syndrome: a closer look at the growing epidemic and its associated pathologies. Obes Rev.

[4] Bartha JL, González-Bugatto F, Fernández-Macías R, González-González NL, Comino-Delgado R, Hervías-Vivancos B (2008). Metabolic syndrome in normal and complicated pregnancies. Eur J Obstet Gynecol Reprod Biol.

[5] Jayasinghe IU, Agampodi TC, Dissanayake AK, Srimantha SM, Agampodi SB (2022). Comparison of global definitions of metabolic syndrome in early pregnancy among the Rajarata Pregnancy Cohort participants in Sri Lanka. Sci Rep.

[6] Barker DJP, Osmond C (1986). Infant mortality, childhood nutrition, and ischaemic heart disease in England and Wales. Lancet.

[7] Rinaudo P, Wang E (2012). Fetal programming and metabolic syndrome. Annu Rev Physiol.

[8] Gluckman PD, Lillycrop KA, Vickers MH, Pleasants AB, Phillips ES, Beedle AS (2007). Metabolic plasticity during mammalian development is directionally dependent on early nutritional status. Proc Natl Acad Sci U S A.

[9] Armitage JA, Khan IY, Taylor PD, Nathanielsz PW, Poston L (2004). Developmental programming of the metabolic syndrome by maternal nutritional imbalance: how strong is the evidence from experimental models in mammals?. J Physiol.

[10] Hochberg Z, Feil R, Constancia M, Fraga M, Junien C, Carel JC (2011). Child health, developmental plasticity, and epigenetic programming. Endocr Rev.

[11] Sun J, Mei H, Xie S, Wu L, Wang Y, Mei W (2019). The interactive effect of pre-pregnancy overweight and obesity and hypertensive disorders of pregnancy on the weight status in infancy. Sci Rep.

[12] Hu Z, Tylavsky FA, Han JC, Kocak M, Fowke JH, Davis RL (2019). Maternal Metabolic factors during pregnancy predict early childhood growth trajectories and obesity risk: the CANDLE Study. Int J Obes.

[13] Logan KM, Gale C, Hyde MJ, Santhakumaran S, Modi N (2017). Diabetes in pregnancy and infant adiposity: Systematic review and meta-analysis. Arch Dis Child Fetal Neonatal Ed.

[14] Shrestha D, Workalemahu T, Tekola-Ayele F (2019). Maternal dyslipidemia during early pregnancy and epigenetic ageing of the placenta. Epigenetics.

[15] Heerwagen MJR, Miller MR, Barbour LA, Friedman JE (2010). Maternal obesity and fetal metabolic programming: a fertile epigenetic soil. Am J Physiol Regul Integr Comp Physiol.

[16] McCloskey K, Ponsonby A-L, Collier F, Allen K, Tang MLK, Carlin JB (2018). The association between higher maternal pre-pregnancy body mass index and increased birth weight, adiposity and inflammation in the newborn. Pediatr Obes.

[17] Frias AE, Grove KL (2012). Obesity: a transgenerational problem linked to nutrition during pregnancy. Semin Reprod Med.

[18] Dabelea D, Crume T (2011). Maternal environment and the transgenerational cycle of obesity and diabetes. Diabetes.

[19] Geraghty AA, Alberdi G, O’Sullivan EJ, O’Brien EC, Crosbie B, Twomey PJ (2016). Maternal blood lipid profile during pregnancy and associations with child adiposity: findings from the ROLO study. PLoS One.

[20] Lemas DJ, Brinton JT, Shapiro ALB, Glueck DH, Friedman JE, Dabelea D (2015). Associations of maternal weight status prior and during pregnancy with neonatal cardiometabolic markers at birth: the healthy start study. Int J Obes.

[21] McAninch D, Bianco-Miotto T, Gatford KL, Leemaqz SY, Andraweera PH, Garrett A (2020). The metabolic syndrome in pregnancy and its association with child telomere length. Diabetologia.

[22] Grieger JA, Bianco-Miotto T, Grzeskowiak LE, Leemaqz SY, Poston L, McCowan LM (2018). Metabolic syndrome in pregnancy and risk for adverse pregnancy outcomes: a prospective cohort of nulliparous women. PLoS Med.

[23] Migda M, Migda MS, Migda B, Krzyzanowska P, Wender-Ozegowska E (2016). Components of metabolic syndrome in the first trimester of pregnancy as predictors of adverse perinatal outcome. Ginekol Pol.

[24] Grieger JA, Leemaqz SY, Knight EJ, Grzeskowiak LE, McCowan LM, Dekker GA (2021). Relative importance of metabolic syndrome components for developing gestational diabetes. Arch Gynecol Obstet.

[25] Habibi N, Mousa A, Tay CT, Khomami MB, Patten RK, Andraweera PH (2022). Maternal metabolic factors and the association with gestational diabetes: a systematic review and meta-analysis. Diabetes Metab Res Rev.

[26] Pathirana MM, Lassi ZS, Ali A, Arstall MA, Roberts CT, Andraweera PH (2021). Association between metabolic syndrome and gestational diabetes mellitus in women and their children: a systematic review and meta-analysis. Endocrine.

[27] Migda M, Migda MS, Migda B, Wender-Ozegowska E (2017). Maternal first trimester parameters in the prediction of excessive fetal growth in pregnant women with metabolic syndrome. J Physiol Pharmacol.

[28] Lear SA, Gasevic D (2020). Ethnicity and metabolic syndrome: implications for assessment, management and prevention. Nutrients.

[29] Misra A, Khurana L (2009). The metabolic syndrome in South Asians: Epidemiology, determinants, and prevention. Metab Syndr Relat Disord.

[30] Agampodi TC, Wickramasinghe ND, Prasanna RIR, Irangani MKL, Banda JMS, Jayathilake PMB, et al. The Rajarata Pregnancy Cohort (RaPCo): study protocol. BMC Pregnancy Childbirth. 2020;20(1):1–13. 10.1186/s12884-020-03056-x PMC7318435 32586287

[31] Alberti KGMM, Eckel RH, Grundy SM, Zimmet PZ, Cleeman JI, Donato KA (2009). Harmonizing the metabolic syndrome a joint interim statement of the international diabetes federation task force on epidemiology and prevention; national heart, lung, and blood Institute; American heart association; world heart federation. International A Circulation.

[32] World Health Organization. Diagnostic Criteria and Classification of Hyperglycaemia First Detected in Pregnancy. World Health Organization; 2013. p. 1–63. 24199271

[33] Jayasinghe IU, Koralegedara IS, Agampodi SB (2022). Early pregnancy hyperglycaemia as a significant predictor of large for gestational age neonates. Acta Diabetol.

[34] World Health Organization WPR, Obesity IAF the S of. The Asia-Pacific perspective: Redifining Obesity and its Treatment. 2000;

[35] Villar J, Altman D, Purwar M, Noble J, Knight H, Ruyan P (2013). The objectives, design and implementation of the INTERGROWTH-21st Project. BJOG An Int J Obstet Gynaecol.

[36] Fahed G, Aoun L, Zerdan MB, Allam S, Zerdan MB, Bouferraa Y (2022). Metabolic syndrome: updates on pathophysiology and management in 2021. Int J Mol Sci.

[37] Rochlani Y, Pothineni NV, Kovelamudi S, Mehta JL (2017). Metabolic syndrome: pathophysiology, management, and modulation by natural compounds. Ther Adv Cardiovasc Dis.

[38] Vrijkotte TGM, Krukziener N, Hutten BA, Vollebregt KC, Van Eijsden M, Twickler MB (2012). Maternal lipid profile during early pregnancy and pregnancy complications and outcomes: The ABCD Study. J Clin Endocrinol Metab.

[39] Adank MC, Benschop L, Kors AW, Peterbroers KR, Smak Gregoor AM, Mulder MT (2020). Maternal lipid profile in early pregnancy is associated with foetal growth and the risk of a child born large-for-gestational age: a population-based prospective cohort study. BMC Med.

[40] Pazhohan A, Rezaee Moradali M, Pazhohan N (2019). Association of first-trimester maternal lipid profiles and triglyceride-glucose index with the risk of gestational diabetes mellitus and large for gestational age newborn. J Matern Neonatal Med.

[41] Chatzi L, Plana E, Daraki V, Karakosta P, Alegkakis D, Tsatsanis C (2009). Metabolic syndrome in early pregnancy and risk of preterm birth. Am J Epidemiol.

[42] Kutchi I, Chellammal P, Akila A (2020). Maternal obesity and pregnancy outcome: in perspective of new Asian Indian guidelines. J Obstet Gynaecol India.

[43] Ghodke B, Pusukuru R, Mehta V (2017). Association of lipid profile in pregnancy with preeclampsia, gestational diabetes mellitus, and preterm delivery. Cureus.

[44] Wani K, Sabico S, Alnaami AM, Al-Musharaf S, Fouda MA, Turkestani IZ (2020). Early-pregnancy metabolic syndrome and subsequent incidence in gestational diabetes mellitus in Arab women. Front Endocrinol (Lausanne).

[45] Chatzi L, Plana E, Pappas A, Alegkakis D, Karakosta P, Daraki V (2009). The metabolic syndrome in early pregnancy and risk of gestational diabetes mellitus. Diabetes Metab.

[46] Wiznitzer A, Mayer A, Novack V, Sheiner E, Gilutz H, Malhotra A (2009). Association of lipid levels during gestation with preeclampsia and gestational diabetes mellitus: a population-based study. Am J Obstet Gynecol.

[47] Zhu H, He D, Liang N, Lai A, Zeng J, Yu H (2020). High serum triglyceride levels in the early first trimester of pregnancy are associated with gestational diabetes mellitus: a prospective cohort study. J Diabetes Investig.

[48] Adank MC, Benschop L, Peterbroers KR, Smak Gregoor AM, Kors AW, Mulder MT (2019). Is maternal lipid profile in early pregnancy associated with pregnancy complications and blood pressure in pregnancy and long term postpartum?. Am J Obstet Gynecol.

[49] Vernini JM, Nicolosi BF, Arantes MA, Costa RA, Magalhães CG, Corrente JE (2020). Metabolic syndrome markers and risk of hyperglycemia in pregnancy: a cross-sectional cohort study. Sci Rep.

[50] Jorquera G, Echiburú B, Crisosto N, Sotomayor-Zárate R, Maliqueo M, Cruz G (2020). Metformin during pregnancy: effects on offspring development and metabolic function. Front Pharmacol.

[51] Chatzakis C, Cavoretto P, Sotiriadis A (2021). Gestational diabetes mellitus pharmacological prevention and treatment. Curr Pharm Des.

