# Supplementary material for: Early pregnancy metabolic syndrome and risk for adverse pregnancy outcomes: findings from Rajarata Pregnancy Cohort (RaPCo) in Sri Lanka
Source: BMC Pregnancy Childbirth. 2023 Apr 5;23:231. doi: 10.1186/s12884-023-05548-y (PMC10074348; doi:10.1186/s12884-023-05548-y)
Supplement: Supplementary file 1 — Additional file 1. [file 12884_2023_5548_MOESM1_ESM.pdf]

STROBE Statement for the cohort study "Early pregnancy metabolic syndrome and risk for adverse pregnancy outcomes: Findings from Rajarata Pregnancy Cohort (RaPCo) in Sri Lanka"

	Item No	Recommendation	Section and paragraph no.
**Title and abstract**	1	(*a*) Indicate the study's design with a commonly used term in the title or the abstract	Title
		(*b*) Provide in the abstract an informative and balanced summary of what was done and what was found	Abstract
**Introduction**			
Background/rationale	2	Explain the scientific background and rationale for the investigation being reported	Introduction, Para 1,2,3
Objectives	3	State specific objectives, including any prespecified hypotheses	Introduction Para 4
**Methods**			
Study design	4	Present key elements of study design early in the paper	Methods – Study design and population Para 1
Setting	5	Describe the setting, locations, and relevant dates, including periods of recruitment, exposure, follow-up, and data collection	Methods – Study design and population Para 1
Participants	6	(*a*) Give the eligibility criteria, and the sources and methods of selection of participants. Describe methods of follow-up	Methods – Study design and population - Para 2 Methods- Outcome data collection – Para 1
		(*b*) For matched studies, give matching criteria and number of exposed and unexposed	
Variables	7	Clearly define all outcomes, exposures, predictors, potential confounders, and effect modifiers. Give diagnostic criteria, if applicable	Methods – Baseline data collection – Para 1, 2, 3 Outcome data collection – Para 2 Data analysis – Para 2, 3, 4
Data sources/ measurement	8*	For each variable of interest, give sources of data and details of methods of assessment (measurement). Describe comparability of assessment methods if there is more than one group	Methods- Baseline data collection – Para 1

			Outcome data collection – Para 1, 2, 3<br>In both groups with and without exposure, same variables were analysed.
Bias	9	Describe any efforts to address potential sources of bias	Methods – Data analysis - Para 2, 4
Study size	10	Explain how the study size was arrived at	Methods – Study design and population – Para 3
Quantitative variables	11	Explain how quantitative variables were handled in the analyses. If applicable, describe which groupings were chosen and why	Methods – Data analysis – Para 1, 2, 3, 4
Statistical methods	12	(*a*) Describe all statistical methods, including those used to control for confounding<br>(*b*) Describe any methods used to examine subgroups and interactions<br>(*c*) Explain how missing data were addressed<br>(*d*) If applicable, explain how loss to follow-up was addressed<br>(*e*) Describe any sensitivity analyses	Methods – Data analysis – Para 1, 2, 3, 4<br>Methods – Data analysis – Para 4<br>Methods – Data analysis – Para 2<br>Results – Figure 01<br>Methods – Outcome data collection – Para 2<br>-
**Results**			
Participants	13*	(a) Report numbers of individuals at each stage of study—eg numbers potentially eligible, examined for eligibility, confirmed eligible, included in the study, completing follow-up, and analysed<br>(b) Give reasons for non-participation at each stage<br>(c) Consider use of a flow diagram	Results - Figure 1, Pregnancy outcome- Para 1
Descriptive data	14*	(a) Give characteristics of study participants (eg demographic, clinical, social) and information on exposures and potential confounders<br>(b) Indicate number of participants with missing data for each variable of interest	Results- Cohort characteristics – Para 1, 2, Table 1, Table 2<br>Data of main baseline variables – None missing<br>Outcome data missing – Results – Pregnancy outcome – Para 1

		(c) Summarise follow-up time (eg, average and total amount)	Results – Pregnancy outcome – Para 1
Outcome data	15*	Report numbers of outcome events or summary measures over time	Results – Pregnancy outcome – Para 1 Additional File 2
Main results	16	(*a*) Give unadjusted estimates and, if applicable, confounder-adjusted estimates and their precision (eg, 95% confidence interval). Make clear which confounders were adjusted for and why they were included	Results – table 1, 2, 3, 4, 5 Additional File 2. Metabolic parameters and pregnancy outcomes, MetS as a predictor of selected pregnancy outcomes, The completed cohort analysis with t2 GDM/DM
		(*b*) Report category boundaries when continuous variables were categorized	Table 1
		(*c*) If relevant, consider translating estimates of relative risk into absolute risk for a meaningful time period	-
Other analyses	17	Report other analyses done—eg analyses of subgroups and interactions, and sensitivity analyses	Results - The completed cohort analysis with t2 GDM/DM, Figure 2
**Discussion**			
Key results	18	Summarise key results with reference to study objectives	Discussion – Para 1
Limitations	19	Discuss limitations of the study, taking into account sources of potential bias or imprecision. Discuss both direction and magnitude of any potential bias	Discussion – Para 6
Interpretation	20	Give a cautious overall interpretation of results considering objectives, limitations, multiplicity of analyses, results from similar studies, and other relevant evidence	Discussion – Para 2, 3, 4, 5
Generalisability	21	Discuss the generalisability (external validity) of the study results	Discussion – Para 6
**Other information**			
Funding	22	Give the source of funding and the role of the funders for the present study and, if applicable, for the original study on which the present article is based	Funding

*Give information separately for exposed and unexposed groups. :- For this cohort, the details of both exposed and unexposed groups are provided in the above mentioned sections.
