# Supplementary material for: Early pregnancy metabolic syndrome and risk for adverse pregnancy outcomes: findings from Rajarata Pregnancy Cohort (RaPCo) in Sri Lanka
Source: BMC Pregnancy Childbirth. 2023 Apr 5;23:231. doi: 10.1186/s12884-023-05548-y (PMC10074348; doi:10.1186/s12884-023-05548-y)
Supplement: Supplementary file 2 — Additional file 2. [file 12884_2023_5548_MOESM2_ESM.pdf]

Summary of main results of the study cohort

Baseline characteristics			
Number of study participants for cohort		2326	
Prevalence of MetS		5.9% (n= 137, 95% CI- 5.0-6.9)	
Prevalence of MetS by revised definition		7.7% (n= 180, 95% CI- 6.7-8.9)	
Loss to follow-up in the cohort		2.8% (n= 64)	
Pregnancy Outcome Assessment			
	Study cohort (n=2326)	With MetS (n=137)	Without MetS (n=2189)
Number of singleton live births	2027 (87.1%)	118 (86.1%)	1909 (87.2%)
Gestational age at delivery (weeks)[*]	38.0 (3)	37.8 (2)	38.8 (1.6)
Gestational weight gain (kg) [*]	9.4 (4.4)	7.9 (3.8)	9.5 (4.4)
Neonatal birth weight (g) [*]	2938.9 (451.5)	3051.8 (533.9)	2931.8 (444.9)
Neonatal birth weight centile[*]	41.83 (29.1)	53.4 (30.2)	41.1 (28.9)
Associations of first trimester MetS with pregnancy outcomes			
**a) Cumulative incidence of the outcomes per 1000 pregnant women**			
	Study cohort (n=2326)	With MetS (n=137)	Without MetS (n=2189)
LGA neonates	61.5	146	57
SGA neonates	151	66	156
PTB	98.5	146	96
MC	95	117	94
**b) Relative risks for the outcomes studied**			
	MetS (RR, 95% CI)	Revised MetS (RR, 95% CI)	
LGA neonates	**2.59** (1.65-3.93)	**2.9** (2.0-4.27)	
SGA neonates	**0.41** (0.29-0.78)	**0.4** (0.25-0.74)	
PTB	1.51 (0.98-2.30)	**1.5** (1.04-2.21)	
MC	1.23 (0.76-1.98)	1.4 (0.97-2.14)	
**c) Risk predictors of adverse outcomes after adjustment for confounders**			
	Significant predictor	*Odds Ratio (95% CI)*	
LGA neonates	Obesity	2.29 (1.47-3.55)	
	T1 hyperglycemia	2.06 (1.40-3.03)	
	Age	1.05 (1.01-1.09)	
SGA neonates	MetS	0.48 (0.24-0.99)	
	Primi gravida	1.80 (1.29-2.52)	
	Underweight	1.56 (1.16-2.10)	
	Obesity	0.67 (0.52-0.87)	
MC	Age	1.05 (1.01-1.08)	
PTB	None		

MetS- Metabolic Syndrome, LGA- Large for gestational age, SGA- Small for gestational age, PTB- Pre term birth, MC- Miscarriages, CI- Confidence interval, T1- First trimester, RR- Relative risk. [*]Mean values are presented with standard deviation
